# Responsibility

**Published:** 1868-10-01

**Authors:** 


### *Responsibility.*

It would seem unnecessary to state that the editor of this, or any other periodical, does not, from his position, hold himself responsible for the views or statements of correspondents. The editor's business is to make his paper a medium of communication between the different members of the profession, and thus stimulate healthful investigation and disseminate the most advanced ideas. *Good faith* is what is demanded of all parties. The present editor proposes always to permit large latitude of opinion and expression to correspondents, only claiming that the usual courtesies of gentlemanly intercourse be preserved. Thus, if any one of our readers disagrees with either correspondents or editors, all he has to do is to put down his reasons therefor in respectable English, transmit it to the JOURNAL, and if it is not too long, and is to the point, we will immortalize the opinion in our pages.

